# Binding of Pramipexole to Extrastriatal Dopamine D_2_/D_3_ Receptors in the Human Brain: A Positron Emission Tomography Study Using ^11^C-FLB 457

**DOI:** 10.1371/journal.pone.0017723

**Published:** 2011-03-09

**Authors:** Kenji Ishibashi, Kenji Ishii, Keiichi Oda, Hidehiro Mizusawa, Kiichi Ishiwata

**Affiliations:** 1 Positron Medical Center, Tokyo Metropolitan Institute of Gerontology, Tokyo, Japan; 2 Department of Neurology and Neurological Science, Graduate School, Tokyo Medical and Dental University, Tokyo, Japan; Chiba University Center for Forensic Mental Health, Japan

## Abstract

The purpose of this study was to determine the binding sites of pramipexole in extrastriatal dopaminergic regions because its antidepressive effects have been speculated to occur by activating the dopamine D_2_ receptor subfamily in extrastriatal areas. Dynamic positron emission tomography (PET) scanning using ^11^C-FLB 457 for quantification of D_2_/D_3_ receptor subtype was performed on 15 healthy volunteers. Each subject underwent two PET scans before and after receiving a single dose of pramipexole (0, 0.125, or 0.25 mg). The study demonstrated that pramipexole significantly binds to D_2_/D_3_ receptors in the prefrontal cortex, amygdala, and medial and lateral thalamus at a dose of 0.25 mg. These regions have been indicated to have some relation to depression and may be part of the target sites where pramipexole exerts its antidepressive effects.

## Introduction

Pramipexole is a dopamine D_2_ receptor (D_2_R) subfamily agonist. It was introduced for treating motor symptoms in patients with idiopathic Parkinson's disease (PD) [1] and has been shown to be effective by various clinical trials [2], [3]. In addition, various studies have recently found antidepressive effects of pramipexole not only in patients with PD complicated by depressive state [4], [5], [6], [7], but also in depressive patients without parkinsonian symptoms [8], [9], [10], [11], [12], [13], [14], [15], [16]. Its antidepressive effects have been also shown in animal experiments [17], [18].

The D_2_R subfamily consists of D_2_, D_3_, and D_4_ receptor subtypes [19]. Pramipexole is active mainly at D_2_ and D_3_ receptors, and compared with other dopamine agonists, it is unique in that the binding affinity for D_3_ receptors is higher than that for D_2_ receptors [20], [21], [22], [23]. Kvernmo et al. reported that binding affinities (inhibition constant; K_i_) for cloned human D_2_ and D_3_ receptors of pramipexole were 3.9 and 0.5 nmol/L, respectively [23]. The distribution of D_3_ receptors in the brain is different from that of D_2_ receptors [24], [25], [26], [27], [28], [29]. Compared with D_2_ receptors, D_3_ receptors are predominantly located in extrastriatal regions including the mesolimbic dopamine system involved in mood and behavior. On the other hand, although both D_2_ and D_3_ receptors in the striatum are much more abundant than those in other regions, the D_2_ receptor density is higher than the D_3_ receptor density in the striatum. Stimulation of D_2_ and D_3_ receptors appears to induce different effects.

There are growing evidences that D_3_ receptors may play a role in the pathogenesis of depression because of their pharmacology and distribution in the brain [26], [30], [31], although the exact mechanism remains unknown. The mechanism of antidepressive effects and extrastriatal binding sites of pramipexole are also unknown, and no study has investigated this issue. These effects have been speculated to occur by means of activation of D_2_R subfamily, especially the D_3_ receptor subtype, in the mesolimbic dopamine system [1]. Therefore, we aimed to determine the binding sites of pramipexole in the extrastriatal dopaminergic regions by using ^11^C-FLB 457 positron emission tomography (PET) scanning for quantification of D_2_/D_3_ receptors in extrastriatal brain regions. In addition, we discussed whether the regional sites occupied by pramipexole may be target sites where pramipexole exerts its antidepressive effects on the basis of previous anatomical and functional reports on depression.

## Materials and Methods

### Subjects

This study protocol was approved by the Ethics Committee of the Tokyo Metropolitan Institute of Gerontology. Written informed consent was obtained from all participants. A total of 15 healthy volunteers (7 men and 8 women; mean age  =  50.2 years, SD = 11.7, range  =  30–77) participated in the study. All subjects underwent two ^11^C-FLB 457 PET scans and magnetic resonance imaging (MRI) of the brain. They were classified into 3 groups according to the dose of pramipexole (0.25, 0.125, or 0 mg). The 5 subjects in the high-dose group (2 men and 3 women, 57.2±12.8 years) received a single oral 0.25 mg dose of pramipexole. Another 5 subjects in the low-dose group (2 men and 3 women, 51.4±9.4 years) received a single oral 0.125 mg dose of pramipexole. The drug was administered between the two PET scans. The other 5 subjects were in the control group (3 men and 2 women, 42.0±6.3 years) and received no medication. Significant difference was not found in age between the 3 groups with one-way ANOVA test. All volunteers were free of any current or past mental disorders, and defined as healthy on the basis of their medical history, the results of their physical and neurological examinations and routine mental health interview performed by a neurologist, and the findings of the MRI. None had been receiving any other medications at the time of this study.

### Doses of pramipexole

Previous studies have shown that administration of more than approximately 1 mg of pramipexole exerts antidepressive effects [4], [5], [6], [7], [8], [9], [10], [11], [12], [13], [14], [15], [16]; compared with these studies, the doses of pramipexole used in our study were low. We chose these low doses to ensure the safety of the participants in this study.

According to unpublished data on file in Nippon Boehringer Ingelheim (Tokyo, Japan), a single administration of pramipexole 0.4 mg caused orthostatic hypotension in an early clinical trial of German volunteers. On the basis of these results, the doses of pramipexole were set at 0.1, 0.2, and 0.3 mg in the Phase one clinical trial of Japanese volunteers, and no one developed more than moderate adverse effects. After oral administration of a single dose of pramipexole 0.1 mg, C_max_, T_max,_ and t_1/2_ were 294.6±46.3 pg/mL, 1.5±0.5 h and 7.71±1.90 h (mean ± SD), respectively. After administration of pramipexole 0.2 mg, the values were 583.2±69.9 pg/mL, 1.4±0.5 h, and 6.36±1.46 h, respectively; after a dose of 0.3 mg, the values were 766.3±88.8 pg/mL, 2.3±1.2 h, and 6.94±1.09 h, respectively. One tablet of pramipexole equals 0.125 mg. Therefore, the doses of pramipexole were set at 0.25 and 0.125 mg in this study.

### ^11^C-FLB 457 PET imaging

Each volunteer participated in two ^11^C-FLB 457 PET scans on the same day—one in the morning and another in the afternoon. Of 15 subjects, 10 were administered with either 0.25 or 0.125 mg of pramipexole after the first PET scan; the second PET scan took place 1–1.5 h later because the concentration of pramipexole in plasma reaches its peak in approximately 1–2 hours as described above.

PET imaging was performed at the Positron Medical Center, Tokyo Metropolitan Institute of Gerontology, with a SET-2400W scanner (Shimadzu, Kyoto, Japan). The spatial resolution was 4.4 mm full width at half maximum in the transverse direction and 6.5 mm full width at half maximum in the axial direction. Images with 50 slices were obtained with a 2×2×3.125-mm voxel size and a 128×128 matrix size. The transmission data were acquired by using a rotating ^68^Ga/^68^Ge rod as a source for attenuation correction. ^11^C-FLB 457 was prepared as described previously [32].

In the first PET experiment, the injected dose, specific activity, and mass of injected ligand were 283±24 MBq, 118±45 GBq/µmol, and 3.0±1.7 µg (mean ± SD), respectively. The respective values in the second PET experiment were 285±19 MBq, 110±38 GBq/µmol, and 3.2±2.0 µg. The time interval between the first and second injections of ^11^C-FLB 457 was 4–4.5 hours. The mass of injected ligand in each second scan was carefully adjusted to that in each first scan because of potential occupancy effects by unlabelled ligand itself [33], [34], and no significant difference was found in the mass of injected ligand as well as the injected dose and specific activity between the first and second scans in each group, using paired Student *t* test.

A dynamic series of decay-corrected PET data acquisition was performed in the 3D mode for 90 minutes starting at the time of the intravenous injection of ^11^C-FLB 457. The frame arrangement was 20 s ×6 frames, 60 s ×2 frames, 180 s ×2 frames, and 300 s ×16 frames.

For the 5 subjects in the high-dose group, arterial blood samples were also obtained. Immediately after the intravenous injection of ^11^C-FLB 457, 18 arterial blood samples were collected at 10-s intervals over 3 min; the next 2 samples were collected at 60-s intervals over 2 min, and the remaining 10 samples were collected at longer intervals, for a total of 30 samples. All samples were manually drawn. Plasma was separated, weighed, and measured for radioactivity with a sodium iodide (Tl) well scintillation counter. Six samples collected at 3, 10, 20, 30, 40, and 60 minutes were further processed by high-performance liquid chromatography to determine the fractions of plasma radioactivity corresponding to unchanged ^11^C-FLB 457 and labeled metabolites, as described previously [32].

### Data analysis

Image manipulations were performed using Dr. View version R2.0 (AJS, Tokyo, Japan) and statistical parametric mapping 2 (SPM2; Functional Imaging Laboratory, London, UK) implemented in MATLAB version 7.0.1 (The MathWorks, Natick, MA). First, individual two dynamic ^11^C-FLB 457 images and MRI images were coregistered. Next, regions of interest (ROIs) were defined over the prefrontal, parietal, lateral temporal and anterior cingulate cortices, medial and lateral parts of the thalamus, amygdala, hippocampus, and cerebellum on the individual coregistered MRI. These ROIs were spatially moved on the corresponding coregistered dynamic ^11^C-FLB 457 images.

^11^C-FLB 457 binding to extrastriatal D_2_/D_3_ receptors was calculated as the binding potential (BP) by the simplified reference tissue model (SRTM) using cerebellum as a reference tissue [35]. BP derived with this method is referred to as BP_ND_SRTM_ (ND: nondisplaceable). For the high-dose group with arterial blood samples, the binding was also analyzed by using the linear graphic analysis by Logan et al. [36]. The slope of the linear phase of the obtained plot corresponds to the total distribution volume (*V*_T_) of the ligand plus the plasma volume. The regional *V*_T_ was determined from the slope, and the BP with this method was calculated as follows using cerebellum as a reference region: BP_ND_Logan_  =  (*V*_T_ on ROI/*V*_T_ on cerebellum) - 1.

D_2_/D_3_ occupancy rate by pramipexole was calculated for each ROI by using the following equation: occupancy rate (%) = 100× (BP at baseline – BP at pramipexole-loading)/BP at baseline. BP at baseline and BP at pramipexole-loading are obtained from first and second PET scans, respectively. Data were expressed as mean ± SD.

### Statistical Analysis

The differences between first and second PET scans were tested by paired Student's *t*-test. Correlations between BP_ND_SRTM_ and BP_ND_Logan_ in high-dose group were assessed by means of linear regression analysis with Pearson's correlation test. *P* values<0.05 were considered statistically significant.

## Results

**Figure 1:**
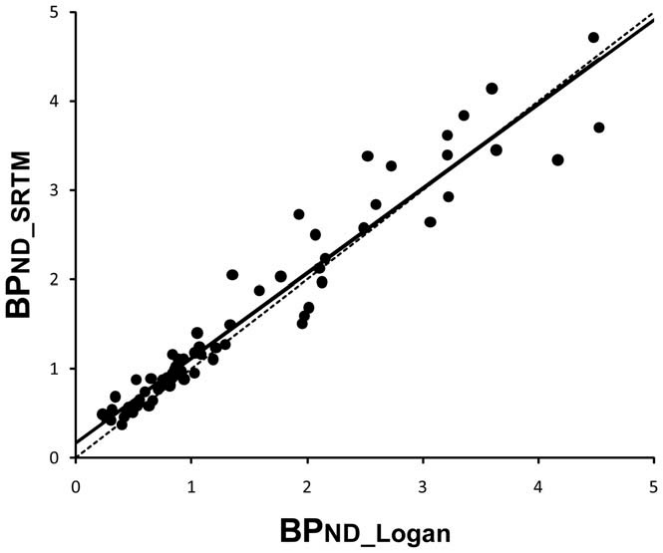
Correlation between BP_ND_Logan_ and BP_ND_SRTM_. The binding potential estimated by Logan plot method and that estimated by the simplified reference tissue model method are represented as BP_ND_Logan_ and BP_ND_SRTM_, respectively. The solid line represents the regression line. Linear correlation is significant (r = 0.97, *P*<0.01, y = 0.95x + 0.17). The dotted line represents the line of “y = x” for reference. ND: nondisplaceable.

For the high-dose group, BP_ND_SRTM_ was found to be significantly correlated with BP_ND_Logan_ (r = 0.97; *P*<0.01) using data of both the first and second experiments, as shown in Figure 1. Although the slope of the regression line was slightly less than one, the BP_ND_SRTM_ and BP_ND_Logan_ had almost a one-to-one relationship.

**Figure 2:**
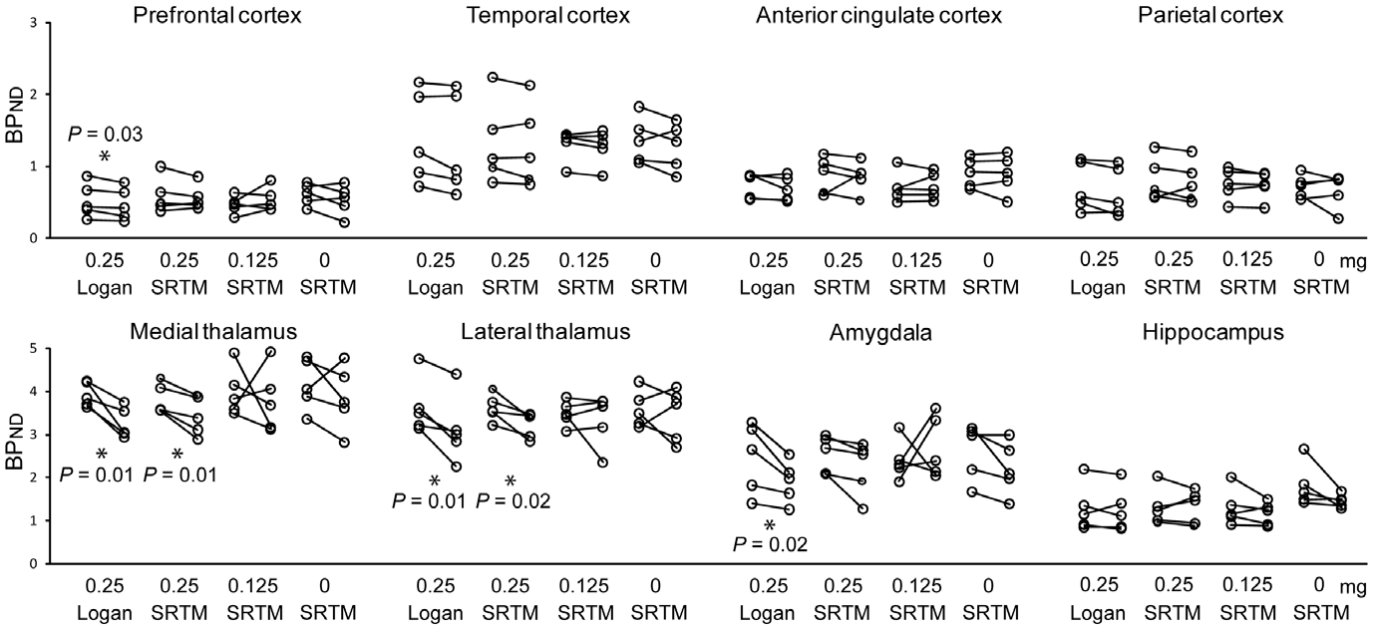
Changes in BP_ND_ between first and second PET scans in each extrastriatal region. For 0.25 mg dose group, each BP_ND_ was estimated by the Logan plot method and simplified reference tissue model method. For 0.125 mg and 0 mg dose groups, each BP_ND_ was estimated only by the simplified reference tissue model method. Pramipexole was orally administered 1–1.5 h before second PET scanning at doses of 0.25 mg, 0.125 mg. Each *P* value was estimated by paired Student's *t*-test between first and second PET scans. Significant differences were found only in the high-dose group (* *P*<0.05). BP: binding potential, ND: nondisplaceable.

Each regional BP_ND_ of first and second PET scans for each dose of pramipexole is shown in Figure 2. After administration of pramipexole 0.25 mg, BP_ND_Logan_ in the prefrontal cortex (*P* = 0.03, *t* = 3.15), medial (*P* = 0.01, *t* = 4.56) and lateral (*P* = 0.01, *t* = 3.78) thalamus, and amygdala (*P* = 0.02, *t* = 3.32) and BP_ND_SRTM_ in medial (*P* = 0.01, *t* = 4.51) and lateral (*P* = 0.02, *t* = 3.33) thalamus decreased significantly. D_2_/D_3_ occupancy rates estimated with BP_ND_Logan_ in the prefrontal cortex, medial and lateral thalamus, and amygdala were 10.3%±6.8%, 16.7%±6.9%, 14.9%±8.9%, and 20.4%±8.6%, respectively. Occupancy rates estimated with BP_ND_SRTM_ in the medial and lateral thalamus were 10.3%±5.0% and 10.8%±6.4%, respectively. No significant difference was found in occupancy rates estimated with either BP_ND_Logan_ or BP_ND_SRTM_ between the medial and lateral thalamus, using paired Student *t* test. In the low-dose group with 0.125 mg of pramipexole and in the control group, there was no significant correlation between BP_ND_SRTM_ of the two PET scans in all regions.

**Figure 3:**
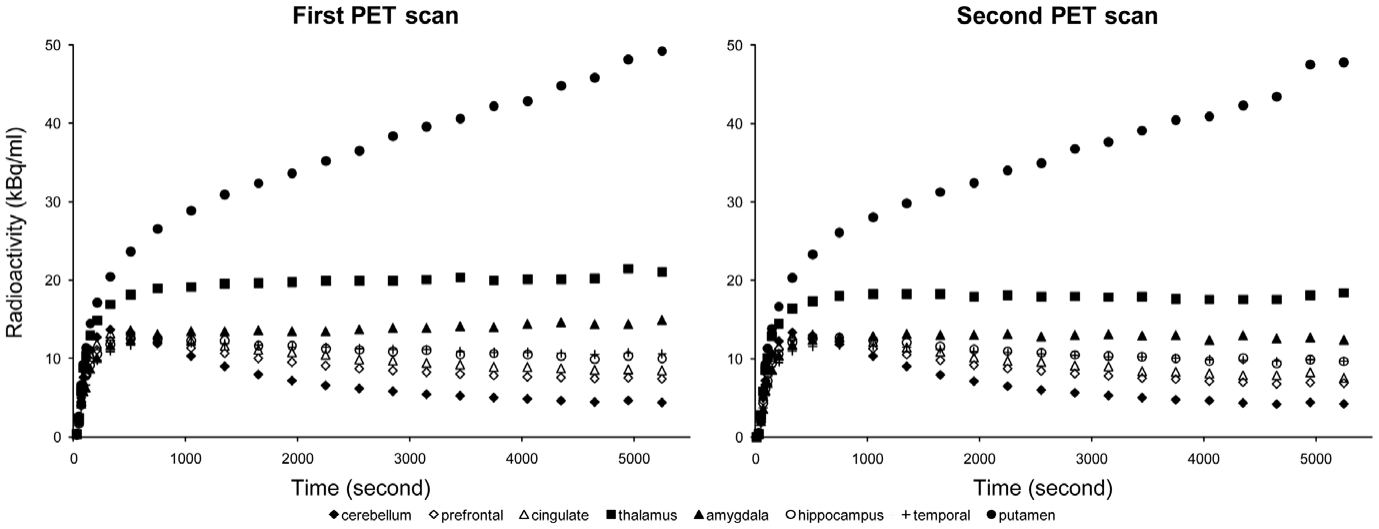
Average time-activity curves of representative regions of five subjects before (left) and after (right) administration of pramipexole 0.25 mg. Each point was normalized to the radioactivity of 185 MBq. The time-activity curve of the putamen is displayed for reference. Thalamus (▪) represents the entire thalamus.

The time-activity curves of representative regions before and after administration of pramipexole 0.25 mg are displayed in Figure 3. Visually, the radioactivity levels of the putamen, entire thalamus and amygdala seemed to decrease after administration of pramipexole 0.25 mg. On the other hand, the radioactively level of the cerebellum seemed mostly unchanged between the first and second scans. Actually, *V*_T_ on the cerebellum estimated by Logan plot method in the first and second PET scans was 4.55±0.68 and 4.67±0.97, respectively, and no significant difference was found between the two.

## Discussion

Pramipexole is a synthetic aminobenzothiazole derivative with selective actions mainly on D_2_ and D_3_ receptors, and it binds with the highest affinity to D_3_ receptors [20], [21], [22], [23]. In the brain, the distribution of D_3_ receptors is known to be different from that of D_2_ receptors [24], [25], [26], [27], [28], [29], although there are some differences in the relative proportion of D_2_ and D_3_ receptors between the previous studies. The D_2_ binding sites are widely detected with the highest concentration found in the striatum, followed by the nucleus accumbens, external segment of the globus pallidus, substantia nigra and ventral tegmental area. The distribution of D_3_ receptors is relatively restricted and D_3_ binding sites are enriched in the amygdala, nucleus accumbens, ventral striatum, substantia nigra, anteroventral nucleus of the thalamus and internal segment of the globus pallidus. Thus, D_2_ or D_3_ binding sites are located in the synapse of the afferent structures as well as the neurons of the efferent structures such as substantia nigra and ventral tegmental area. D_2_ or D_3_ receptors in the efferent structures are thought to act as autoreceptors, which could play an important role in regulating the activity of dopaminergic neurons [24], [29].

On the other hand, in this study, the BP_ND_ in the substantia nigra or ventral tegmental area could not be quantified with reliability, because the structures were too small for usual ROI analysis of the dynamic data on the basis of the resolution of the PET scanner and the number of subjects was relatively small. For the same reasons, the ROIs were drawn over not each small nucleus but medial and lateral parts of the thalamus although the thalamus is known to have a great deal of regional heterogeneity in D_2_ and D_3_ expression. Also, the BP_ND_ in the striatal and its closely neighbor regions such as the ventral striatum and globus pallidus could not be quantified using ^11^C-FLB 457 because a long time more than a few hours is needed for reaching equilibration in the striatum [37]. Based on the distribution of D_2_ and D_3_ receptors and the technical matters as described above, we investigated the binding sites of pramipexole especially in the limbic system, thalamus and cortical regions. To our knowledge, this is the first in vivo study that has investigated the relationship between a dopamine agonist and its binding sites in extrastriatal regions.

This study showed that a single dose of pramipexole 0.25 mg decreased BP_ND_Logan_ significantly in the prefrontal cortex, amygdala, and thalamus. The mesolimbic pathway begins in the ventral tegmental area of the midbrain and projects to the limbic areas, including the nucleus accumbens in the ventral striatum, amygdala, and hippocampus; it also projects to the cortical areas, including the prefrontal and cingulate cortices. The latter cortical pathway is called as the mesocortical pathway. Both pathways are known to be involved in the depressive state [38], [39], [40], [41]. Indeed, the amygdala is important for emotional processing and its functional abnormalities are associated with depression [42], [43], [44], and frontal cortical dopamine function has been reported to be involved in depression [45], [46], [47]. In the mesolimbic pathway, D_2_ and D_3_ binding sites are predominant in the hippocampus and amygdala, respectively [24], [25], [27], and this difference may be one of the reasons that significant decrease of BP_ND_Logan_ was not found in the hippocampus. The mesocortical pathway also has D_3_ receptors as well as D_2_ receptors [27], [29], although there is no detail report on the relative proportion of D_2_ and D_3_ receptors in that area. The thalamic dopaminergic system has been recently identified; this system is speculated to have a prominent role in depression, especially in regard to emotion, attention, cognition, and complex somatosensory and visual processing [44], [48], [49], [50]. Although this study could not find the significant difference between medial and lateral parts of the thalamus, D_3_ binding sites are relatively abundant and especially tend to be concentrated along the midline in the thalamus, while D_2_ binding sites are more homogeneously distributed [24], [27]. On the basis of the relationship between the occupied sites by pramipexole, the distribution of D_2_ and D_3_ receptors and previous anatomical and functional reports on depression, it is reasonable to suggest that pramipexole may exert its antidepressive effects by activating D_2_R subfamily, especially the D_3_ receptor subtype, in these regions (prefrontal cortex, amygdala, and thalamus).

With regard to the BP_ND_SRTM_ method, after administration of a single dose of pramipexole 0.25 mg, binding in the medial and lateral thalamus decreased significantly as shown in the BP_ND_Logan_ method, and bindings in both the prefrontal cortex and amygdala showed the tendency to decrease without significant difference. On the other hand, there was no significant difference between first and second PET scans in both the low-dose group and the control group. On the basis of the difference between the high-dose group and the other two groups, we speculate that the effects of pramipexole may be dose dependent, although it is impossible to confirm this finding only with our data. Despite the high correlation between BP_ND_Logan_ and BP_ND_SRTM_ methods as shown in Figure 1, the discrepancies between the both methods found in the high-dose group could be explained by the interindividual and intraindividual variability of each analytical method [51], [52], [53]. However, BP_ND_Logan_ estimated with artery blood samples should be regarded as more reliable and accurate than BP_ND_SRTM_ estimated without artery blood samples, and the findings for the prefrontal cortex and amygdala, where only the BP_ND_Logan_ method showed significance, were considered to be meaningful.

Vilkman et al. conducted a test-retest analysis of ^11^C-FLB 457 PET scanning with 7 healthy volunteers (mean age ± SD = 29.0±6.9) and suggested that coefficient of variation (COV) of each extrastriatal region in BP_ND_Logan_ and BP_ND_SRTM_ methods was about 20% and the reproducibility of both methods was good [51]. Consistent with Vilkman et al., our results of the control group corresponding to a test-retest analysis showed no significant difference in BP_ND_SRTM_ of each region between first and second PET scans. In the control group, the COV of each region between the first and second experiments was similar, and the ranges of COV in the first and second experiments were 19.3%–33.9% and 16.3%–38.5%, respectively. Thus, the reproducibility of the BP_ND_SRTM_ method in this study was good. Compared with previous studies [51], [52], [53], the COV in our control group was somewhat larger. A possible explanation for this difference could be given by following two reasons. One is the relatively smaller number of subjects, and the other is the relatively larger variability in age because D_2_R subfamily in each extrastriatal region is known to show age-related decline [54], [55].

In vitro brain homogenate binding studies have demonstrated that D_2_R subfamily exists in two affinity states, i.e., high and low affinity states [56], [57], [58]. The high affinity state is thought to represent the functional state, and agonists bind preferentially to D_2_R subfamily in the high affinity state, while antagonists have equal affinity for D_2_R subfamily in the high and low affinity states. In vivo competition studies between endogenous dopamine and a labeled agonist or antagonist ligand estimated the percentage of high affinity state to be about 60–70% [59], [60]. On the other hand, some recent in vivo studies indicated that most D_2_R subfamily is in the high affinity state at living conditions because the binding of exogenous unlabeled agonist to D_2_R subfamily in high or low affinity states could not be differentiated with either a labeled agonist or antagonist ligand [61], [62]. Thus, the accurate proportion of the two states remains controversial. In this study, relatively low D_2_/D_3_ occupancy rates by pramipexole were mainly due to low dose of pramipexole. However, based on the two states theory, another reason may be because D_2_/D_3_ occupancy rates by agonist pramipexole were estimated by antagonist ligand ^11^C-FLB 457.

One of the drawbacks of this study may be that we used the cerebellum as a reference region, in order to gain smaller variability and better reproducibility for the analysis of the PET data, compared with the two-tissue compartment four-rate constant model [51]. Asselin et al. reported that using the cerebellum as a reference region could lead to underestimation of BP_ND_ and occupancy rate [63]. However, we showed no statistical difference in *V*_T_ on the cerebellum estimated by Logan plot method before and after administration of pramipexole 0.25 mg, and at least our data, especially in the high-dose group, would be appropriate for the purpose of confirming the extrastriatal effects of a dopamine agonist. Other drawbacks of this study may be that we collected arterial blood samples only from the high-dose group, the number of subjects was relatively small and a dose of pramipexole was relatively low for safety, as described previously.

In conclusion, we demonstrated that pramipexole binds to D_2_/D_3_ receptors in the prefrontal cortex, amygdala, and medial and lateral thalamus. These regions have been indicated to have some relation to depression and may be part of the target sites where pramipexole exerts its antidepressive effects.
